# MiR‐409‐3p regulates cell proliferation and tumor growth by targeting E74‐like factor 2 in osteosarcoma

**DOI:** 10.1002/2211-5463.12177

**Published:** 2017-01-27

**Authors:** Jun Zhang, Wengen Hou, Jinling Jia, Yilei Zhao, Bin Zhao

**Affiliations:** ^1^ Department of OrthopaedicsThe First Affiliated Hospital of Xinxiang Medical CollegeWeihuiHenanChina

**Keywords:** E74‐like factor 2, miR‐409‐3p, osteosarcoma, tumor suppressor

## Abstract

Recent evidence has shown that miR‐409‐3p was down‐regulated in several types of cancer, including osteosarcoma. However, the potential role of miR‐409‐3p in osteosarcoma remains largely unknown. In the present study, we showed that overexpression of miR‐409‐3p in osteosarcoma cells inhibited cell proliferation *in vitro* and suppressed tumor growth *in vivo*, and the restoration of miR‐409‐3p promoted G1/S cell cycle arrest and induced cell apoptosis. Additionally, E74‐like factor 2 (ELF2) was recognized as a new target of miR‐409‐3p by dual‐luciferase reporter assay. Restoration of ELF2 rescued the inhibitory effect of miR‐409‐3p on cell proliferation in osteosarcoma cells. Moreover, ELF2 was up‐regulated in osteosarcoma tissues and negatively associated with miR‐409‐3p levels. Taken together, our findings collectively indicate that miR‐409‐3p may be a tumor suppressor in osteosarcoma and may serve as a promising therapeutic target for osteosarcoma.

**Abbreviations**
3′‐UTR: 3′‐untranslated regions
CCK‐8: Cell Counting Kit‐8
DMEM: Dulbecco's modified Eagle's medium
ELF2: E74‐like factor 2
HRP: horseradish peroxidase
miRNA: microRNA
NC: negative control
PI: propidium iodide
qRT‐PCR: real‐time quantitative PCR
RIPA: radio immunoprecipitation assay

Osteosarcoma is an aggressive malignant mesenchymal neoplasm among adolescents. Despite the significantly developed therapeutic strategies for osteosarcoma, patients with lung metastases have a worse prognosis and their 5‐year survival rate is far lower 1. Therefore, to understand the molecular pathogenesis of this disease is essential to design new, effective therapeutic strategies to improve patient survival.

MicroRNAs (miRNAs) are a class of endogenous, noncoding, single‐stranded small regulatory RNA molecules, which are highly conserved in evolution 2. miRNAs affect transcriptional and post‐transcriptional regulation of gene expression by binding to the 3′‐untranslated regions (3′‐UTRs) of target mRNAs. Thereby suppressing protein translation 3. miRNA dysregulation is emerging as an important contributor to many human diseases including cancer 4. miR‐409‐3p was first reported being expressed by embryonic stem cells 5. Down‐regualtion of miR‐409‐3p has been observed in numerous human malignancies, including gastric cancer 6, 7, prostate cancer 8, bladder cancer 9, lung adenocarcinoma 10, colorectal cancer 11, 12 as well as breast cancer 13, 14. Additionally, studies using cancer cell lines found that miR‐409‐3p regulates cell proliferation, invasion, and metastasis through regulating multiple target genes, thus serving as a tumor suppressor.

Recently, miR‐409‐3p was reported to be decreased in osteosarcoma tissues, and it was also suggested to be negatively correlated with metastasis in osteosarcoma patients 15. Furthermore, miR‐409‐3p could suppress osteosarcoma cell migration and invasion by targeting catenin‐δ1 15. However, the role of miR‐409‐3p in osteosarcoma growth remains elusive.

In this study, we explored the potential role of miR‐409‐3p in osteosarcoma growth and its underlying mechanisms. Our results showed that miR‐409‐3p significantly inhibited osteosarcoma cell proliferation *in vitro* and suppressed tumor growth *in vivo*. Besides, miR‐409‐3p promoted G1/S cell cycle arrest and induced cell apoptosis in osteosarcoma cells. Additionally, E74‐like factor 2 (ELF2) maybe a novel target of miR‐409‐3p. Together, our data indicate that miR‐409‐3p may be a tumor suppressor in osteosarcoma by targeting ELF2.

## Materials and methods

### Human osteosarcoma specimens

The study was approved by the Research Ethics Committee of The First Affiliated Hospital of Xinxiang Medical College. A total of 36 human osteosarcoma tissues and paired normal bone tissues were obtained between June 2013 and June 2014 from the Department of Orthopaedics, The First Affiliated Hospital of Xinxiang Medical College. Samples were snap frozen and stored at −80 °C until analyzed. Written informed consent was obtained from each patient according to the guidelines from the fields of medical ethics.

### Mice, cell lines, and cell culture

A total of 20 male nude BALB/c mice aged 6–8 weeks and weighing 20–22 g were purchased from Charles River Laboratories (Beijing, China), and maintained in specific pathogen‐free conditions. The experimental procedures were approved by the Laboratory Animal Care Committee at Xinxiang Medical College. All animals received care depending on the Guide for the Care and Use of Laboratory Animals (NIH, Bethesda, MD). Human osteosarcoma cell lines, MG63, SaOS‐2, U2OS, and G292, human normal osteoblast cells NHOst, and human fetal osteoblastic cell line hFOB 1.19 were all purchased from American Type Culture Collection (ATCC, Rockville, MD, USA). Cells were cultured in DMEM (Gibco, Grand Island, NY, USA) supplemented with 10% FBS and maintained at 37 °C in a humidified incubator containing 5% CO_2_.

### Cell transfection

The miR‐409‐3p mimics, miR‐409‐3p inhibitor, micrON® agomiR‐409‐3p, and negative control (NC) were designed and synthesized by RiboBio (Guangzhou, China). Cell transfection was performed as indicated previously 16. Briefly, 2 × 10^5^ MG63 and SaOS‐2 cells were plated in one well of a six‐well plate in 1 mL of complete medium without antibiotics. Cells were transfected with miR‐409‐3p mimics, miR‐409‐3p inhibitor, or NC using HiPerFect Transfection Reagent (Qiagen, Hilden, Germany) when they reached 80% confluence. The mixture was added to cells at a final concentration of 100 nm. After transfection for about 4–6 h, the cultured medium was discarded, and cells were sustained in Dulbecco's modified Eagle's medium (DMEM) with 10% FBS for an additional 48 h.

### Cell viability assay

Twenty‐four hours after transfection, cells were harvested and plated in triplicate in 200 μL of medium in a 96‐well plates at 2 × 10^3^ cells per well. Cell proliferation was assessed every 24 h from day 1 to day 4 using CCK‐8 assay. Briefly, 10 μL of CCK‐8 solution was added to each well and they were incubated at 37 °C and 5% CO_2_ for 4 h. The absorbance at 490 nm was measured using a microplate reader (Bio‐Tek Company, Winooski, VT, USA). The experiment was independently performed three times.

### Cell cycle analysis

Cell cycle was measured using propidium iodide (PI) staining. Briefly, 24 h post‐transfection, cells were harvested, washed with PBS, and fixed in ice‐cold 70% ethanol overnight. Cells were then washed with ice‐cold PBS, resuspended in 500 μL of a PI master mix containing 50 μg·mL^−1^ PI (Sigma, St. Louis, MO, USA) and 100 μg·mL^−1^ RNAse A (Sigma) diluted in PBS and incubated for 30 min in the dark at 37 °C. Cell cycle was measured using BD FACSCalibur flow cytometer (BD Bioscience, San Diego, CA, USA). The gating was carried out manually. The gates for the G0/G1, S, and G2/M phases were set manually in the histogram for the PI area, to ensure that the peak of the G0/G1 region was detected at half of the value of the peak for the G2/M phase.

### Cell apoptosis assay

Cells were plated at the same density than for the cell cycle assay. Twenty‐four hours post‐transfection, cells were harvested, washed with PBS, and were resuspended in 100 μL of 1 × binding buffer, 5 μL of Annexin V antibody conjugated with FITC (BD Pharmingen, San Diego, CA, USA) were added and incubated for 15 min in the dark at room temperature. Then, 200 μL of binding buffer and 5 μL of a 1 mg·mL^−1^ stock solution of PI were added to the cells and they were analyzed within the next hour by FACSCalibur. Data were analyzed using flowjo Software (FlowJo, Ashland, OR, USA). Apoptotic cells were defined as Annexin V^+^ PI^−^.

### *In vivo* tumor growth assay

Xenografts were generated with the osteosarcoma cell lines MG63 and SaOS‐2 in immunocompromised mice. Briefly, transfected 5 × 10^6^ MG63 or SaOS‐2 cells were mixed in 60 μL of cold matrigel (BD Bioscience) and injected subcutaneously into the flank region of nude mice (*n* = 5 per group). Xenografts were left to grow for up to 5 weeks. Tumor growth was monitored with calipers every 5 days, tumor volume was calculated by the following formula: tumor volume = 0.5 × width^2^ × length. At day 35 after tumor injection, tumors were harvested, weighed, and photographed.

### Plasmid construction and Dual‐luciferase reporter assay

Wild‐type (WT) or Mutant (Mut) 3′‐UTR sequence of ELF2 containing miR‐409‐3p‐binding site was cloned into pMIR‐REPOR Luciferase miRNA Expression Reporter Vector (Ambion Life Technologies, Carlsbad, CA, USA). Mixture of pMIR‐REPOR‐ELF2‐WT or pMIR‐REPOR‐ELF2‐Mut (500 ng) and 50 nm miR‐409‐3p mimics or NC were cotransfected into MG63 or SaOS‐2 cells using Lipofectamine 2000 (Invitrogen, Carlsbad, CA, USA). Luciferase activities were measured using the Dual‐Luciferase Reporter Assay System (Promega, Madison, WI, USA) 48 h after transfection, according to the manufacturer's protocol. Firefly luciferase activity was normalized for transfection efficiency using the corresponding Renilla luciferase activity. Primers for ELF2 3′ UTR were 5′‐ACCATGGACTTCAGGCTGTT‐3′ (forward) and 5′‐GGTCCCTTTCGATCCTTTTT‐3′ (reverse).

### RNA extraction and real‐time quantitative PCR

The mRNA and miRNA were extracted from tissues and cell lines using miRNeasy extraction Kit (Qiagene, Shanghai, China) as previously described 16. RNA concentration and quality were measured using a NanoDrop spectrophotometer (Thermo Scientific, Waltham, MA, USA). cDNA was synthesized from mRNA using PrimeScript RT reagent kit with gDNA Eraser (TaKaRa, Tokyo, Japan). The amplification step was performed in a Microamp optical 96‐well plate on an ABI 7500 fast real‐time PCR system (Applied Biosystems, Foster City, CA, USA). The following gene‐specific primers were used in this study: Stem‐loop RT primer for miR‐409‐3p was: 5′‐GTCGTATCCAGTGCGTGTCGTGGAGTCGGCAATTGCACTGGATACGACTGGGGA‐3′. 5′‐GCGAATGTTGCTCGGTGA‐3′ and reverse 5′‐GTGCAGGGTCCGAGGT‐3′ for miR‐409‐3p; forward, 5′‐AAACTGTAGTGGAGGTGTCAACT‐3′ and reverse 5′‐CATGGCTATCTGGTGATGTTGG‐3′ for ELF2; forward, 5′‑AGAGCCTGTGGTGTCCG‑3′ and reverse, 5′‐CATCTTCAAAGCACTTCCCT‐3′ for internal control U6 small nuclear RNA; forward, 5′‐ACAACTTTGGTATCGTGGAAGG‐3′ and reverse, 5′‐GCCATCACGCCACAGTTTC‐3′ for GAPDH. The relative expression of genes was quantified using of comparative *C*_t_ method (ΔΔ*C*_t_) relative to the appropriate housekeeping gene.

### Protein extraction and western blotting

Protein was extracted from cells using lysis buffer containing radio immunoprecipitation assay (RIPA) buffer and protease inhibitor (Beyotime Biotechnology, Shanghai, China). The concentration of the protein lysate was assessed using a bicinchoninic acid protein assay kit (Beyotime Biotechnology). Twenty micrograms of lysate was denatured at 100 °C for 5 min with 7.5 μL of loading buffer, and then separated by 10% SDS/PAGE, proteins were transferred onto polyvinylidene fluoride membranes (Millipore Corporation, Bedford, MA, USA) and blocked for 1 h in 5% bovine milk diluted in TBST. The membrane was then incubated with the primary antibodies to ELF2 (1 : 500; clone 224C4a; Abcam, Cambridge, MA, USA) and β‐actin (1 : 2000; Abcam) overnight at 4 °C. The membrane was subsequently washed three times for 15 min in TBST, and incubated for 1 h at room temperature in a dilution of 1 : 2000 of the secondary antibody in 5% milk in TBST. The blotted membrane was incubated for 1 min with a mix of solution A and B of ECL™ Western Blotting Detection Reagents (GE Healthcare, Buckinghamshire, UK) to visualize the bound antibody.

### Immunohistochemistry

Tumor specimens and xenograft tumors were fixed with 10% neutral formalin and embedded in paraffin. Immunostaining was performed on 4‐μm‐thick sections. Briefly, sections were treated with 3% H_2_O_2_ methanol for 10 min and then washed in water and blocked in 5% goat antiserum for 10 min. Next, the tissue sections were incubated overnight at 4°C in 1 : 500 dilutions of rabbit polyclonal to ELF2 (Abcam), or 1 : 100 dilutions of mouse anti‐cleaved caspase‐3 (Cell Signaling). The sections were then incubated with biotinylated secondary antibodies (1 : 2000) for 1 h. Following a washing step with PBS, streptavidin‐horseradish peroxidase was applied. Finally, the sections were developed with diaminobenzidine tetrahydrochloride substrate for 5–10 min, the desired color reaction was observed when monitored with the microscope. All of the slides were counterstained with hematoxylin.

### Statistical analysis

Data were analyzed using spss version 19.0 software (SPSS, Chicago, IL, USA). All data are shown as means ± SD and analyzed by two‐tailed unpaired Student's *t* test. Correlation between miR‐409‐3p levels and ELF2 expression was assessed using Spearman's correlation analysis. Difference was considered significant when *P* < 0.05.

## Results

### miR‐409‐3p suppresses osteosarcoma cell proliferation *in vitro*

**Figure 1:**
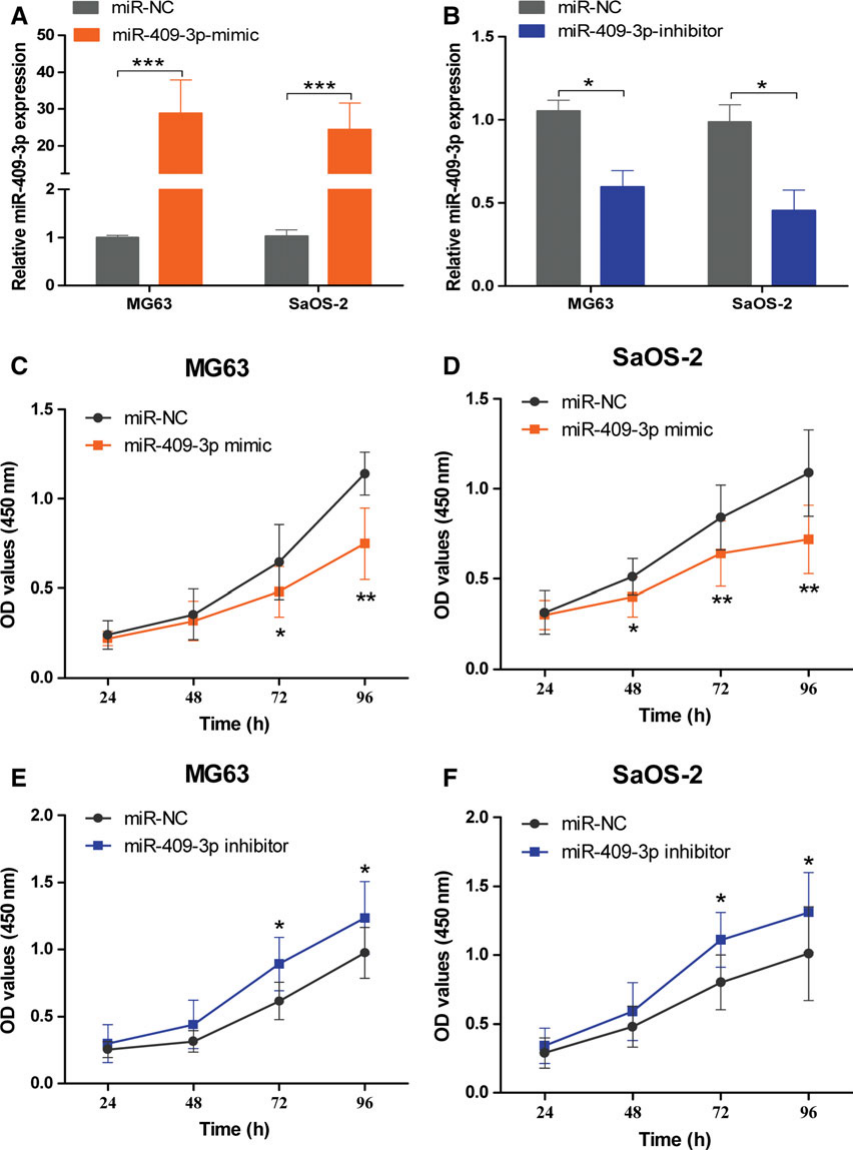
miR‐409‐3p suppresses osteosarcoma cell proliferation *in vitro*. MG63 and SaOS‐2 cells were transfected with hsa‐miR‐409‐3p mimics, miR‐409‐3p inhibitor, or negative control (miR‐NC), respectively. The endogenous levels of miR‐409‐3p were examined by qRT‐PCR (A and B**)**. Time course CCK‐8 assay showed that up‐regulation of miR‐409‐3p decreased cell proliferation compared with cells transfected with the control oligonucleotide (C and D). Knockdown of miR‐409‐3p promoted cell proliferation compared with controls (E and F). Data shown are mean ± SD from three independent experiments. *P < 0.05, **P < 0.01, ***P < 0.001.

MiR‐409‐3p reportedly decreased in osteosarcoma; however, the precise role of miR‐409‐3p in osteosarcoma is still not fully understood. miR‐409‐3p was found down‐regulated in four human osteosarcoma cell lines MG63, SaOS‐2, U2OS, and G292 compared with human normal osteoblast cells NHOst, and human fetal osteoblastic cell line hFOB 1.19 (data not shown), which was consistent with previous study 15. To explore the role of miR‐409‐3p in osteosarcoma, MG63, and SaOS‐2 cells were transiently transfected with miR‐409‐3p mimic or inhibitor to increase or decrease its expression. miR‐409‐3p expression after transfection was shown in Fig. 1A,B. A time course CCK‐8 assay showed that up‐regulation of miR‐409‐3p promoted osteosarcoma cell proliferation (Fig. 1C,D); in contrast, knockdown of miR‐409‐3p had the opposite effect (Fig. 1E,F).

**Figure 2:**
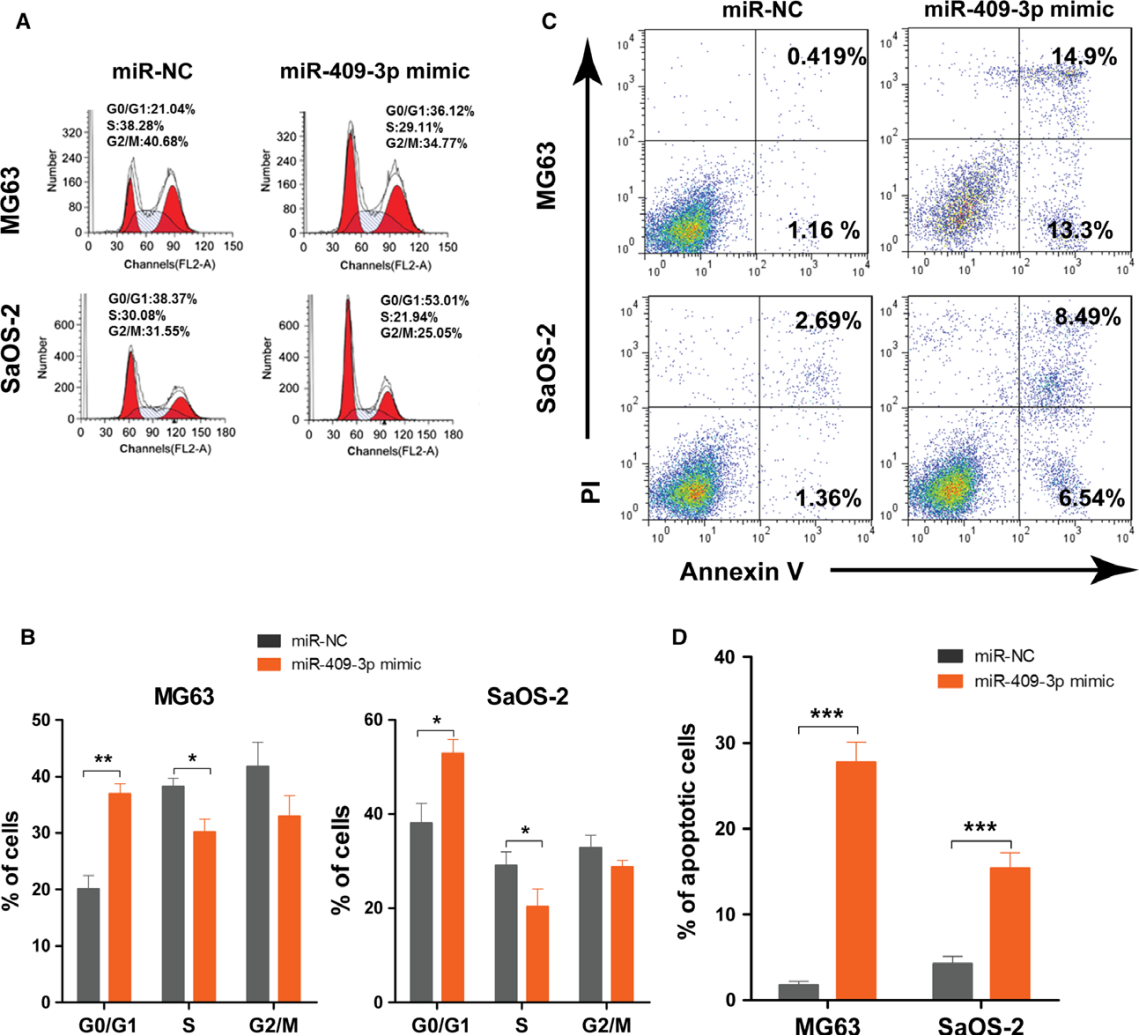
miR‐409‐3p induces cell cycle arrest and apoptosis in osteosarcoma cells. MG63 and SaOS‐2 cells were transfected with hsa‐miR‐409‐3p mimics or negative control (miR‐NC), respectively. Cell cycle distribution was examined by PI staining using flow cytometry. Overexpression of miR‐409‐3p resulted in an increased G0/G1 and reduced S population in miR‐409‐3p mimics‐transfected MG63 and SaOS‐2 cells (A and B). Cell apoptosis was examined by using Annexin V/PI staining. Overexpression of miR‐409‐3p promoted cell apoptosis compared with cells transfected with negative controls in miR‐409‐3p mimics‐transfected MG63 and SaOS‐2 cells (C and D). Data shown are mean ± SD from three independent experiments. *P < 0.05, **P < 0.01, ***P < 0.001.

We then examined the cell cycle distribution. As shown in Fig. 2A,B, overexpression of miR‐409‐3p resulted in an increased G0/G1 and reduced S population in miR‐409‐3p mimic‐transfected MG63 and SaOS‐2 cells; whereas inhibition of miR‐409‐3p promoted cell cycle progression, with increased number of cells in S phase (data not shown). Thus, miR‐409‐3p may suppress cell proliferation through blocking the G1 to S phase transition. Furthermore, cell apoptosis assay indicated that miR‐409‐3p overexpression promoted cell apoptosis compared to cells transfected with NCs in miR‐409‐3p mimic‐transfected MG63 and SaOS‐2 cells (Fig. 2C,D). In contrast, knocking down endogenous miR‐409‐3p significantly inhibited cell apoptosis (data not shown). Taken together, our results suggest that miR‐409‐3p induces cell cycle arrest and cell apoptosis, thus suppressing osteosarcoma cell proliferation *in vitro*.

### miR‐409‐3p inhibits osteosarcoma growth *in vivo*

**Figure 3:**
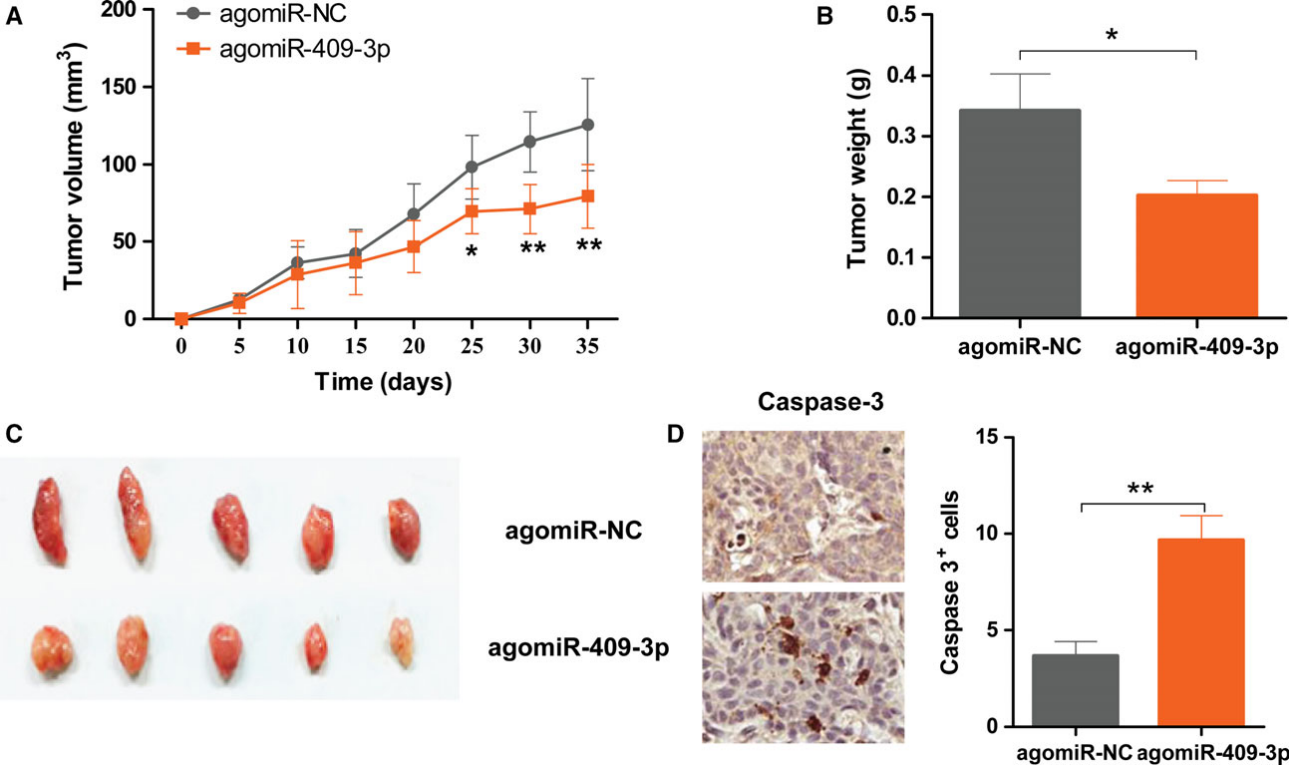
miR‐409‐3p inhibits osteosarcoma growth *in vivo*. MG63 cells stably expressing the miR‐409‐3p were subcutaneously injected into the flank region of nude mice (*n* = 5 per group). Tumor volumes were measured every 5 days up to 5 weeks (A). On day 35, mice were sacrificed, and xenografts were harvested and the tumor weights were measured (B), representative images from five tumors were shown (C), representative images of immunostaining of caspase‐3 of xenografts sections and the quantified apoptotic cell (D). Data shown are mean ± SD from three independent experiments. *P < 0.05, **P < 0.01.

In order to determine if miR‐409‐3p could affect tumor growth *in vivo*, MG63 cells stably expressing the miR‐409‐3p were subcutaneously injected into the flank region of nude mice. As shown in Fig. 3A–C, miR‐409‐3p‐overexpressing xenografts showed smaller tumor volumes and reduced tumor weights compared to the agomiR‐NC xenografts. In addition, we examined the apoptotic cells in the xenografts by cleaved caspase‐3 staining. As shown in Fig. 3D, staining of tumor sections for cleaved caspase‐3 revealed a significant increase in tumor cell apoptosis in miR‐409‐3p‐overexpressing xenografts (*P* < 0.01). Together, our results indicated that miR‐409‐3p inhibits osteosarcoma cell proliferation and tumor growth *in vitro* and *in vivo*.

### miR‐409‐3p directly targets ELF2 in osteosarcoma cells

**Figure 4:**
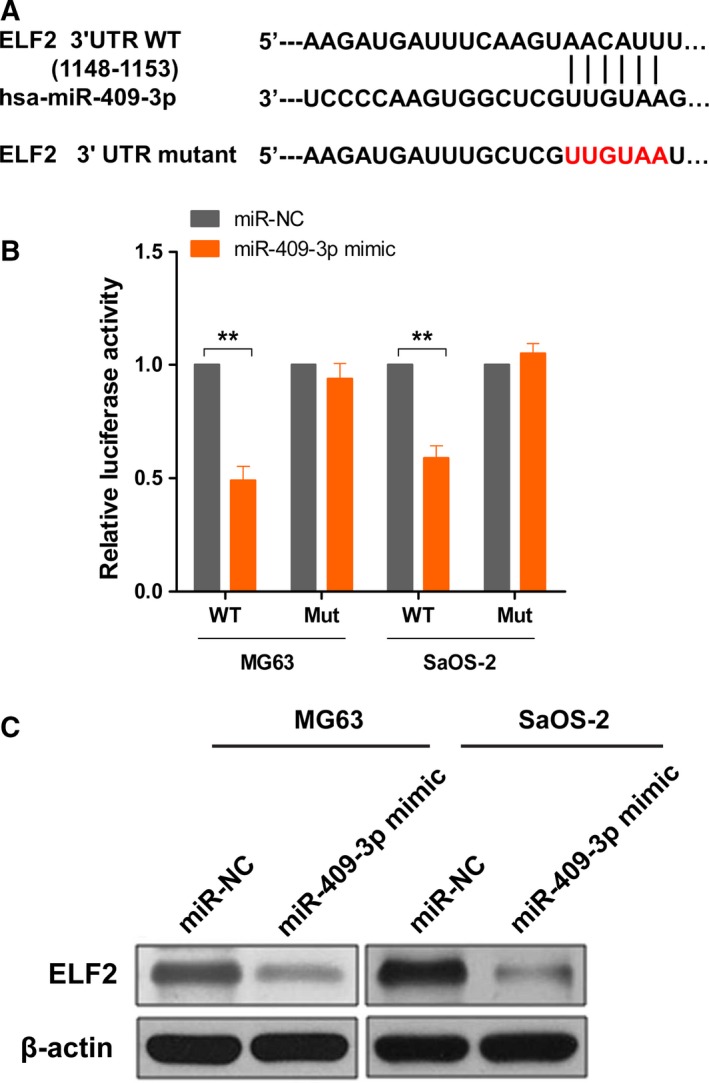
miR‐409‐3p directly targets ELF2 in osteosarcoma cells. Bioinformatics research was performed to find potential targets of miR‐409‐3p using Targetscan and miRanda. Sequence alignment of wild‐type (WT) and mutated (Mut) putative miR‐409‐3p‐binding sites in the 3′‐UTR of ELF2 was shown (A). 3′UTR sequences containing wild‐type or corresponding mutant counterpart of miR‐409‐3p were constructed into the pMIR‐REPORT vector, respectively, and cotransfected with the miR‐409‐3p mimics or NC into MG63 or SaOS‐2 cells. Data showed that miR‐409‐3p remarkably suppressed the relative luciferase activity of the ELF2 ‐3′UTR‐binding site, whereas luciferase activity was not significantly changed in the mutant binding site (B). Overexpression of miR‐409‐3p inhibited the protein levels of ELF2 in both MF63 and SaOS‐2 cells examined by western blot (C). Data shown are mean ± SD from three independent experiments. **P < 0.01.

We then investigated the underlining mechanisms of miR‐409‐3p in osteosarcoma. As shown in Fig. 4A, ELF2 was identified as a potential target of miR‐409‐3p by using Targetscan and miRanda databases. The predicted binding site of miR‐409‐3p was located at the base from positions 1148 to 1153 within the 3′‐UTR of ELF2. We then constructed 3′UTR sequences containing WT or mutant of miR‐409‐3p into the pMIR‐REPORT vector and cotransfected with the miR‐409‐3p mimics or NC into osteosarcoma cells. Dual‐luciferase reporter assay showed that miR‐409‐3p significantly inhibited the relative luciferase activity of the ELF2 ‐3′UTR‐binding site, whereas luciferase activity was not significantly changed in the mutant binding site (Fig. 4B). In addition, we found that the protein levels of ELF2 in both MF63 and SaOS‐2 cells were decreased once miR‐409‐3p was overexpressed (Fig. 4C). These results indicate that miR‐409‐3p directly binds to the 3′‐UTR of ELF2, therefore inhibiting ELF2 expression.

### ELF2 is a critical mediator of miR‐409‐3p in osteosarcoma cells

**Figure 5:**
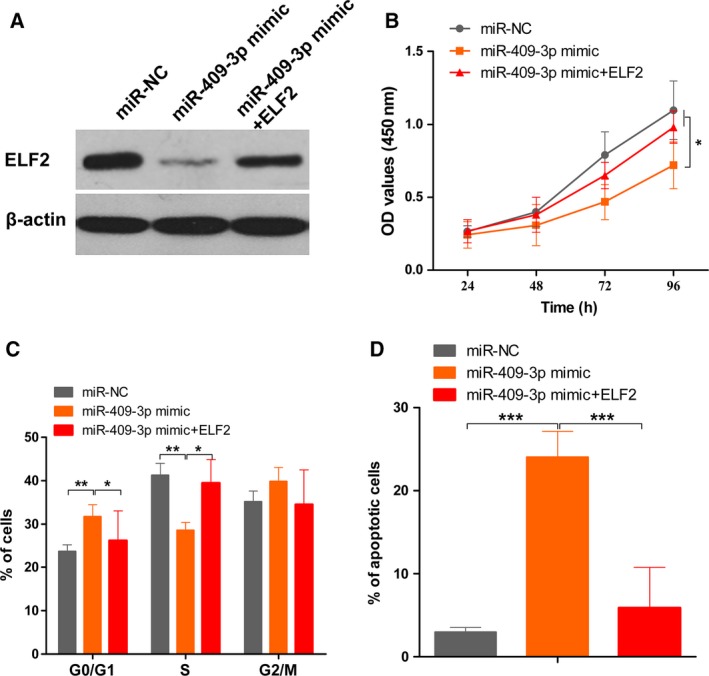
ELF2 is a critical mediator of miR‐409‐3p in osteosarcoma cells. MG63 cells were cotransfected with miR‐409‐3p mimics and ELF2 plasmid (without 3′UTR). The expression levels of ELF2 were examined by western blot (A). The effect of miR‐409‐3p on cell proliferation (B), cell cycle distribution (C), and cell apoptosis (D) was rescued under the condition of overexpression of ELF2. Data shown are mean ± SD from three independent experiments. *P < 0.05, **P < 0.01, ***P < 0.001.

To explore whether the effect of miR‐409‐3p on osteosarcoma growth is mediated by down‐regulating ELF2 expression, we constructed ELF2 plasmid without 3′UTR and cotransfected with miR‐409‐3p mimics into MG63 cells. Transfection of miR‐409‐3p mimics plus ELF2 significantly increased ELF2 expression in MG63 (Fig. 5A). Moreover, CCK‐8 assay and cell cycle assay showed that overexpression of ELF2 in MG63 cells could rescue the inhibitory role of miR‐409‐3p in cell proliferation and G1 to S phase transition (Fig. 5B,C). Cell apoptosis assay demonstrated that overexpression of ELF2 also reversed the promoting effects of miR‐409‐3p on cell apoptosis (Fig. 5D). Hence, our findings suggest that ELF2 may be a direct and functional target of miR‐409‐3p in osteosarcoma cells.

### ELF2 was up‐regulated and inversely correlated with miR‐409‐3p in osteosarcoma tissues

**Figure 6:**
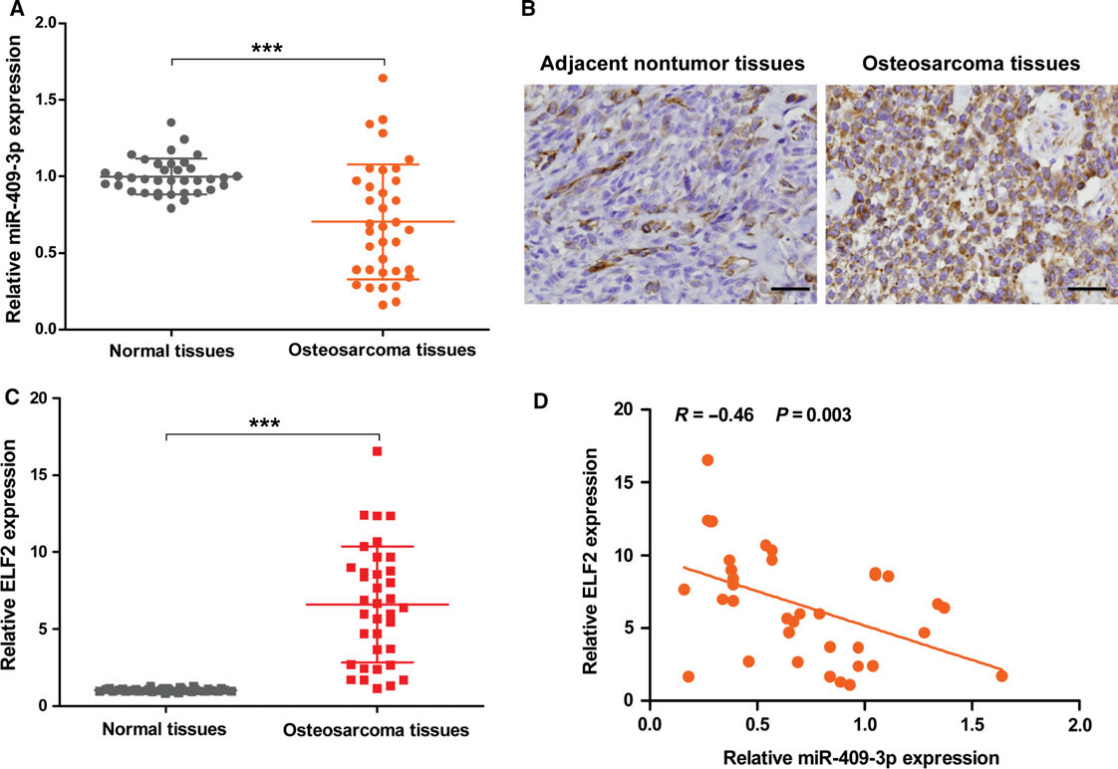
ELF2 was up‐regulated and inversely correlated with miR‐409‐3p in osteosarcoma tissues. The expression of miR‐409‐3p (A) in 36 pairs of osteosarcoma and adjacent normal tissues was examined by qRT‐PCR. (B and C) The expression of and ELF2 in 36 pairs of osteosarcoma and adjacent normal tissues was examined by qRT‐PCR and immunohistochemistry. (D) The correlation between miR‐409‐3p and ELF2 mRNA levels in osteosarcoma tissues was analyzed using Spearman's correlation analysis. Data shown are mean ± SD. ***P < 0.001.

Furthermore, we considered the correlation between of miR‐409‐3p and ELF2. As shown in Fig. 6A, miR‐409‐3p was down‐regulated, whereas the mRNA and protein expression of ELF2 were up‐regulated in osteosarcoma tissues, compared with their paired ajacent nontumor tissues (Fig. 6B,C). Additionally, the results show a significant negative correlation between miR‐409‐3p and ELF2 expression in osteosarcoma tissues (Fig. 6D).

## Discussion

Recently, accumulating evidence has shown that miR‐409‐3p was down‐regulated in a number of tumors, and functions as a tumor suppressor in the tumorigenesis of various types of malignancies by targeting multiple genes. However, little is known about the potential role of miR‐409‐3p in osteosarcoma. In this study, we confirmed that miR‐409‐3p was down‐regulated in osteosarcoma cell lines compared with normal osteoblast and osteoblastic cells. Furthermore, restoration of miR‐409‐3p suppressed cell proliferation, whereas it promoted cell apoptosis *in vitro* through directly targeting the transcript factor ELF2. Our study suggested that miR‐409‐3p may act as a novel tumor suppressor in osteosarcoma.

Previous studies have indicated the deregulated expression of miR‐409‐3p in human cancers, as well as its clinicopathologic significance. For instance, Ma *et al*. reported that miR‐409‐3p levels were down‐regulated and significantly correlated with poor outcomes in patients with breast cancer. Overexpression of miR‐409‐3p inhibited cellular proliferation and suppressed cell migration and invasion by targeting ZEB1 14. At the same time, Zhang *et al*. 13 confirmed that miR‐409‐3p was significantly down‐regulated in breast cancer tissues and cell lines, and miR‐409‐3p suppresses breast cancer cell growth and invasion by targeting Akt1. Similar results were noted in gastric cancer 6, 7, prostate cancer 8, bladder cancer 9, lung adenocarcinoma 10, and colorectal cancer 11, 12. Notably, these results are also in line with our findings showing the tumor suppressor role of miR‐409‐3p in tumor development. However, the function of miR‐409‐3p may seem to vary in different tumors. Josson *et al*. 17 reported in human prostate cancer, that miR‐409‐3p/‐5p was elevated in bone metastatic prostate cancer cell lines and human prostate cancer tissues with higher Gleason scores. Moreover, increased miR‐409‐3p levels correlated with progression‐free survival of patients with prostate cancer. miR‐409‐3p/‐5p promoted tumorigenesis and epithelial‐to‐mesenchymal transition of prostate cancer, suggesting that the role of miR‐409‐3p in cancer progression is complicated. The reasons for this discrepancy need further investigation. However, it is worth noting that circulating miR‐409‐3p was significantly down‐regualted in prostate tumors 8.

MiRNAs regulate a wide range of biological functions through regulating the expression of target genes 3. To classify the underlying mechanism by which miR‐409‐3p functions, we screened the target genes of miR‐409‐3p using bioinformatics analysis. ELF2 was selected as a direct target gene of miR‐409‐3p. However, there was no report about whether miR‐409‐3p could directly target ELF2 in osteosarcoma. ELF2 is an ETS family transcription factor 18. The ETS family of transcription factors plays significant roles in both normal and tumor cells for different biological processes such as cell proliferation, differentiation, apoptosis, migration, invasion, and angiogenesis 19. It was previously reported that ELF2 transactivates valosin‐containing protein (VCP, which was shown to be associated with antiapoptotic function and metastasis via activation of nuclear factor kappa‐B signaling pathway) gene through binding to both upstream and downstream motifs of the conserved region of VCP promoter, and promotes growth and metastasis of breast cancer 20, 21. Recent study showed that overexpression of ELF2 enhanced tumor cell proliferation, and conversely, its knockdown suppressed HepG2 cell growth via p21 and p27 activation 22. In the current study, ELF2 was identified as a direct target of miR‐409‐3p. *In vitro* experiments showed that miR‐409‐3p overexpression reduced endogenous ELF2 levels in osteosarcoma cells. Besides, overexpression of ELF2 also reversed the effects of miR‐409‐3p on cell proliferation, as well as cell apoptosis. However, the underling mechanisms need further investigation. In clinical osteosarcoma tissues, we also found that increased ELF2 levels in osteosarcoma tissues were negatively correlated miR‐409‐3p expression. Taken together, these findings suggest that ELF2 is a direct and functional target of miR‐409‐3p in osteosarcoma.

In conclusion, the results obtained in the current study suggest that miR‐409‐3p may function as a tumor suppressor in osteosarcoma, and ELF2 was a direct target of miR‐409‐3p. Notably, our findings indicate that miR‐409‐3p may be a promising therapeutic target for osteosarcoma treatment.

## Author contributions

BZ and JZ conceived and designed the experiments, analyzed the data, and wrote the manuscript. WGH, LJJ, and YLZ assisted the cell and molecular biology experiments. All authors read and approved the final manuscript.
